# Alterations in regulatory T cells and immune checkpoint molecules in pancreatic cancer patients receiving FOLFIRINOX or gemcitabine plus nab-paclitaxel

**DOI:** 10.1007/s12094-021-02620-x

**Published:** 2021-04-19

**Authors:** L. Sams, S. Kruger, V. Heinemann, D. Bararia, S. Haebe, S. Alig, M. Haas, D. Zhang, C. B. Westphalen, S. Ormanns, P. Metzger, J. Werner, O. Weigert, M. von Bergwelt-Baildon, F. Rataj, S. Kobold, S. Boeck

**Affiliations:** 1 Department of Internal Medicine III and Comprehensive Cancer Center, Grosshadern University Hospital, Ludwig-Maximilians-University of Munich, Marchioninistr. 15, 81377 Munich, Germany; 2 German Cancer Consortium (DKTK), Partner Site Munich, Munich, Germany; 3 Laboratory for Experimental Leukemia and Lymphoma Research (ELLF), Department of Internal Medicine III, Grosshadern University Hospital, Ludwig-Maximilians-University, Munich, Germany; 4 Institute of Pathology, Faculty of Medicine, Ludwig-Maximilians-University, Munich, Germany; 5 Center of Integrated Protein Science Munich (CIPS-M) and Division of Clinical Pharmacology, University Hospital of the Ludwig-Maximilians-University, Munich, Germany; 6 Department of General, Visceral and Transplantation Surgery, Ludwig-Maximilians-University, Munich, Germany

**Keywords:** FOLFIRINOX, Gemcitabine, Nab-paclitaxel, Pancreatic cancer, Immune checkpoints, Regulatory T cells

## Abstract

**Purpose:**

This pilot study aimed on generating insight on alterations in circulating immune cells during the use of FOLFIRINOX and gemcitabine/nab-paclitaxel in pancreatic ductal adenocarcinoma (PDAC).

**Patients and methods:**

Peripheral blood mononuclear cells were isolated before and 30 days after initiation of chemotherapy from 20 patients with advanced PDAC. Regulatory T cells (FoxP3+) and immune checkpoints (PD-1 and TIM-3) were analyzed by flow cytometry and immunological changes were correlated with clinical outcome.

**Results:**

Heterogeneous changes during chemotherapy were observed in circulating T-cell subpopulations with a pronounced effect on PD-1+ CD4+/CD8+ T cells. An increase in FoxP3+ or PD-1+ T cells had no significant effect on survival. An increase in TIM3+/CD8+ (but not TIM3+/CD4+) T cells was associated with a significant inferior outcome: median progression-free survival in the subgroup with an increase of TIM-3+/CD8+ T cells was 6.0 compared to 14.0 months in patients with a decrease/no change (*p* = 0.026); corresponding median overall survival was 13.0 and 20.0 months (*p* = 0.011), respectively.

**Conclusions:**

Chemotherapy with FOLFIRNOX or gemcitabine/nab-paclitaxel induces variable changes in circulating T-cell populations that may provide prognostic information in PDAC.

## Introduction

Pancreatic ductal adenocarcinoma (PDAC) is one of the most lethal cancers of the gastrointestinal tract [1]. Combination chemotherapies with FOLFIRINOX [5-fluorouracil (5-FU), folic acid, irinotecan, oxaliplatin] or gemcitabine/nab-paclitaxel are regarded as standard treatment options for advanced disease [2, 3]. Within the last years, checkpoint inhibitors have revolutionized anti-cancer treatments in multiple malignancies. In contrast, efforts to develop successful immunotherapeutic regimens for PDAC have largely failed so far [4]. Preclinical studies suggested that chemotherapeutic drugs such as gemcitabine, oxaliplatin and 5-FU can have immunogenic effects mediated by the so-called “immunogenic cell death” [5–8]. For assessing the immunogenic status in peripheral blood multiple markers are available: well-known examples are forkhead box P3 (FoxP3), programmed cell death protein 1 (PD-1) and T-cell immunoglobulin and mucin-domain containing-3 (TIM-3). FoxP3 is essential for the differentiation of regulatory T cells (Tregs) and their inhibitory function. Previous studies have suggested a correlation of advanced PDAC stages with an increase in circulating Tregs [9, 10]. PD-1 is expressed on activated T cells and may form—together with its ligand PD-L1—an interaction that inhibits T cells activity [11, 12]. TIM-3 is a regarded as a T-cell exhaustion marker (also expressed on dendritic cells, macrophages and B cells) and an increase of TIM-3 on tumor-infiltrating CD3^+^ T cells was associated with a worse outcome in different tumor types (e.g. stomach or non-small cell lung cancer) [13, 14]. The goal of the present pilot study was thus to gain a better understanding of alterations in circulating T-cell subsets induced by standard chemotherapy regimens.

## Materials and methods

We conducted a prospective, single-center study that included chemotherapy-naïve patients with cytologically or histologically confirmed advanced PDAC who received FOLFIRINOX or gemcitabine/nab-paclitaxel between 2015 and 2017. Blood samples (15 ml heparinized blood, 10 ml serum) were taken at day 1 and 30 of 1st-line chemotherapy. Ficoll density gradient separation was used to isolate peripheral blood mononuclear cells. After short-term storage at − 80 °C, flow cytometry was performed [using a LSR Fortessa flow cytometer (BD Biosciences)] to measure the expression of FoxP3, PD-1 and TIM-3 on CD3/CD4+ and CD3+/CD8+ T cells. To calculate the expression and the change in the expression within the first 30 days, we used the following approach:

Calculation of the expression for the first point in time (before chemotherapy) and the second (during chemotherapy):

$$\frac{{{\text{CD}}4{\text{ or CD}}8{\text{ positive cells}}}}{{{\text{cell count CD}}3}} \times 100 = {\text{Expression }}1{\text{ CD}}4{\text{ o}}.{\text{ CD}}8{\text{ in percent}},$$

$$\frac{{\text{Markerpositive cells}}}{{{\text{cell count CD}}4{\text{ or CD}}8}} \times 100 = {\text{Expression }}1{\text{or }}2{\text{ of a marker in percent}}.$$

Calculation of the change during time:

$${\text{Expression point of time }}2 - {\text{Expression point of time }}1 = {\text{change in the expression in percent}}.$$

The clinical outcome was assessed by the endpoints progression-free survival (PFS) and overall survival (OS). PFS and OS were defined as the time interval from start of chemotherapy to documented disease progression or death from any cause. Patient follow-up was conducted until October 2019. Statistical analyses were performed using GraphPad Prism; time-to-event endpoints were analyzed by the Kaplan–Meier method, survival differences were compared using the log-rank test. This study had approval of the local ethics committee (approval number 284-10).

## Results

Twenty-eight eligible patients were included in the study. For final analysis, patients were excluded if they died or were lost to follow-up before the second blood draw, had an intolerance towards one of the therapeutic agents or an infection (defined by clinical signs, laboratory parameters and/or the use of antimicrobial treatment) within the first 30 days of chemotherapy. Finally, two consecutive blood samples were available for 20 patients (FOLFIRINOX: *n* = 17, gemcitabine/nab-paclitaxel: *n* = 3). Table 1 summarizes the main clinical characteristics of the included patients: median age was 61 years and 19 out of 20 patients suffered from a ductal adenocarcinoma and 1 patient from a mixed adenoneuroendocrine carcinoma of the pancreas.

**Table 1:** Baseline patient characteristics (*n* = 20)

Parameter	
Age [median, years]	61 (range 46–78)
Sex	
Male	13 (65%)
Female	7 (35%)
UICC stage	
III	2 (10%)
IV A	4 (20%)
IV B	14 (70%)
Progression-free survival [median, months]	
FOLFIRINOX	8.0
Gem/nab-Pac	6.0
All patients	8.0
Overall survival [median, months]	
FOLFIRINOX	16.0
Gem/nab-Pac	14.0
All patients	14.0
ECOG	
0–1	19 (95%)
3	1 (5%)
Localization of distant metastases	
Liver	10 (50%)
Non-liver	3 (15%)
Liver and further location	1 (5%)
Histology	
Adenocarcinoma	19 (95%)
MANEC	1 (5%)
Chemotherapy regimen	
FOLFIRINOX	17 (85%)
Gem/nab-Pac	3 (15%)
Surgical therapy prior to inclusion in study	
Yes	10 (50%)
No	10 (50%)

*UICC* Union for International Cancer Control, *ECOG *Eastern Cooperative Oncology Group, *MANEC* mixed adenoneuroendocrine carcinoma

**Fig. 1:**
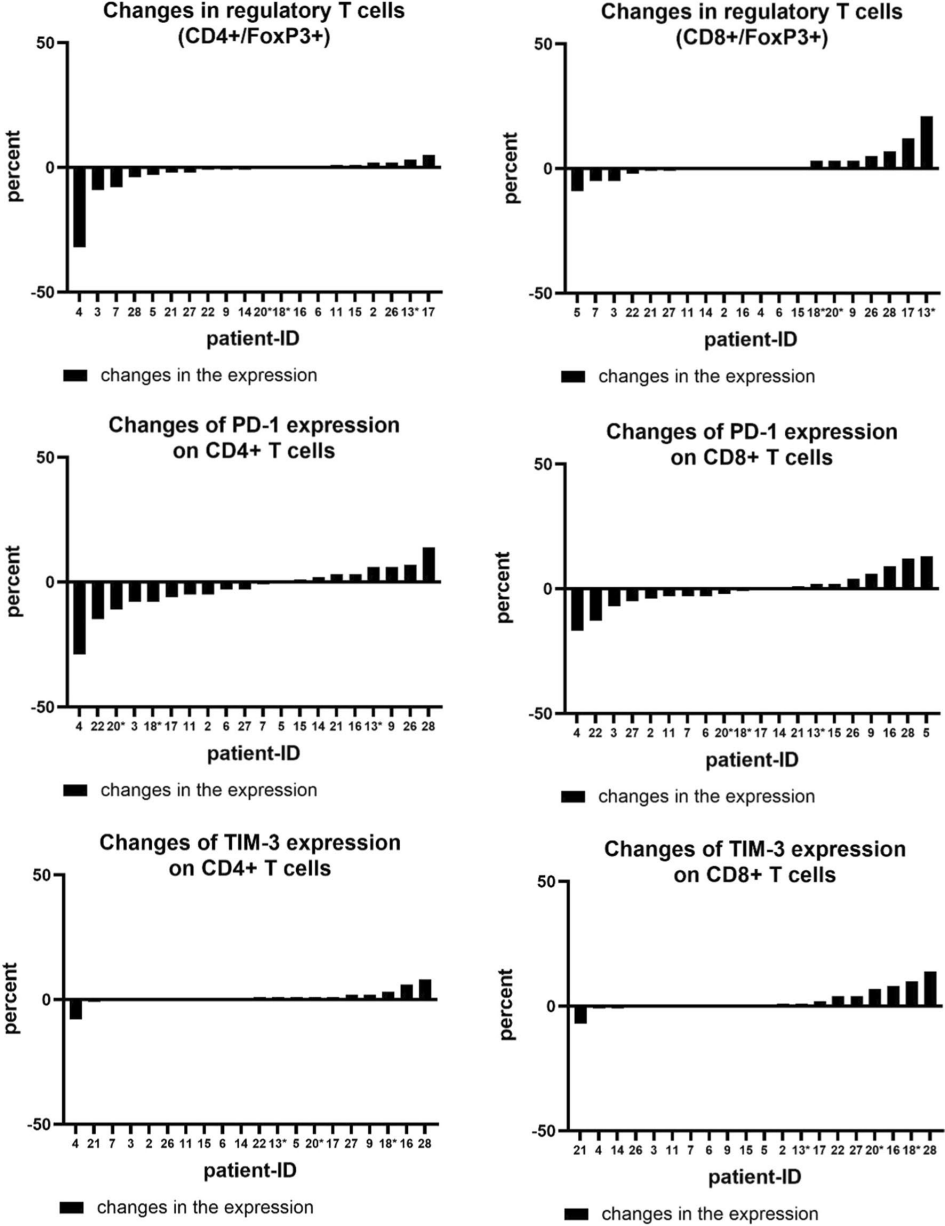
Changes in the expression of regulatory T cells, PD-1+ T cells and TIM-3+ T cells divided in CD4+ and CD8+ subgroups. Patients marked with * received gemcitabine/nab-paclitaxel, all others FOLFIRINOX

**Fig. 2:**
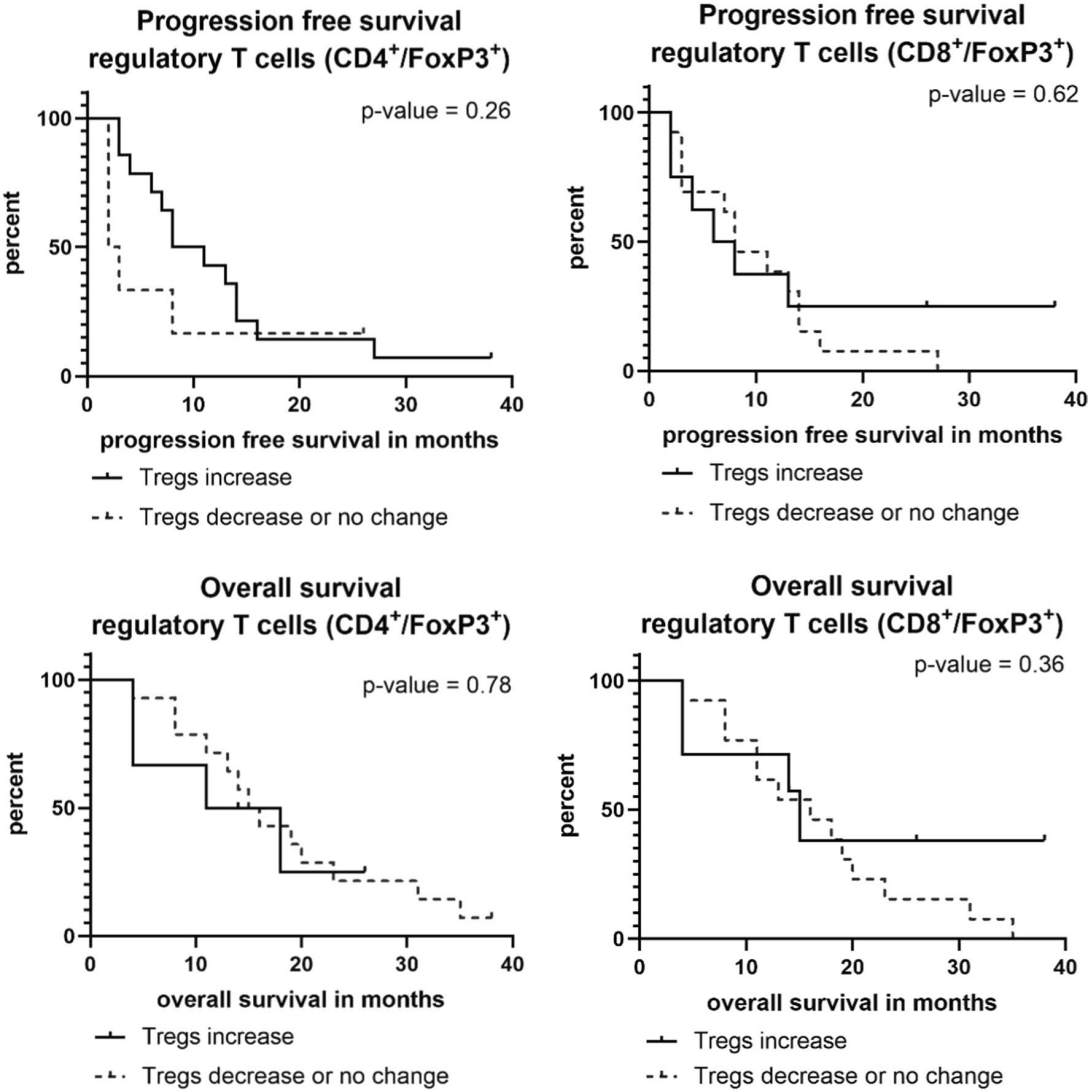
Progression-free and overall survival in patients with an increase or decrease/no change in FoxP3+ Tregs over the first 30 days of chemotherapy

The analysis of the variability within T-cell subsets and the change of expression in Tregs and immune checkpoint molecules during chemotherapy found changes in the expression of all markers: some patients showed a decrease or no change within the first 30 days and some patients showed an increase. Interestingly, we were not able to identify a specific pattern, e. g. an increase in regulatory T cells was not accompanied by an increase in immune checkpoints. Figure 1 illustrates the changes in the expression of T-cell markers during the first 30 days of chemotherapy. Specifically, regulatory T cells and PD-1+ T cells showed significant changes of up to 30% increase or decrease. In a second step, we analyzed whether a change in the expression of a maker correlated with the patient`s clinical outcome. Regarding Tregs, PFS of patients with an increase in CD4+/FoxP3+ T cells was numerically shorter than of those who showed no change or a decrease in the expression (2.5 vs. 9.5 months). This difference, however, was not statistically significant (*p* = 0.263) (Fig. 2). Median OS in patients with an increase in CD4+/FoxP3+ T cells was 14.5 vs. 15.5 months in patients with no change or a decrease (*p* = 0.787). There was no correlation found between a change in CD8+ regulatory T cells and the outcome of patients (Fig. 2).

**Fig. 3:**
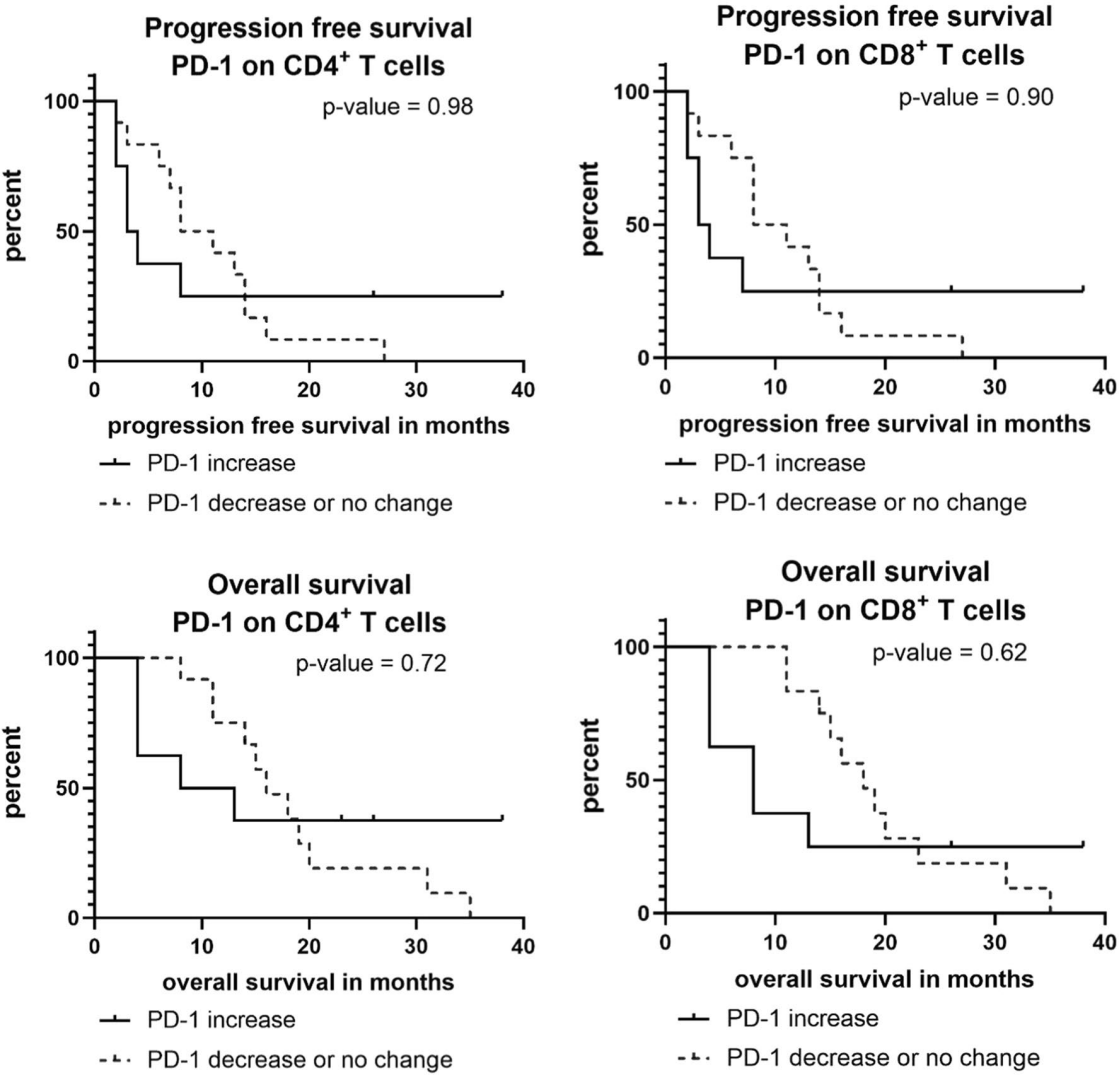
Progression-free and overall survival in patients with an increase or decrease/no change in PD-1+/CD4+ or PD-1+/CD8+ T cells over the first 30 days of chemotherapy

Patients with an increase of PD1+/CD4+ or PD-1+/CD8+ T cells had a short median PFS (3.5 months). Patients without an increase in contrast had a median PFS of 9.5 months (*p* = 0.981 for PD1+/CD4+ and *p* = 0.902 for PD-1+/CD8+ ; see Fig. 3). Median OS was also numerically inferior in patients with an increase in PD-1 expression on CD4+ and CD8+ T cells (for CD4: 10.5 vs. 16.0 months, *p* = 0.725; for CD8: 8.0 vs. 18.0 months, *p* = 0.625; see Fig. 3).

**Fig. 4:**
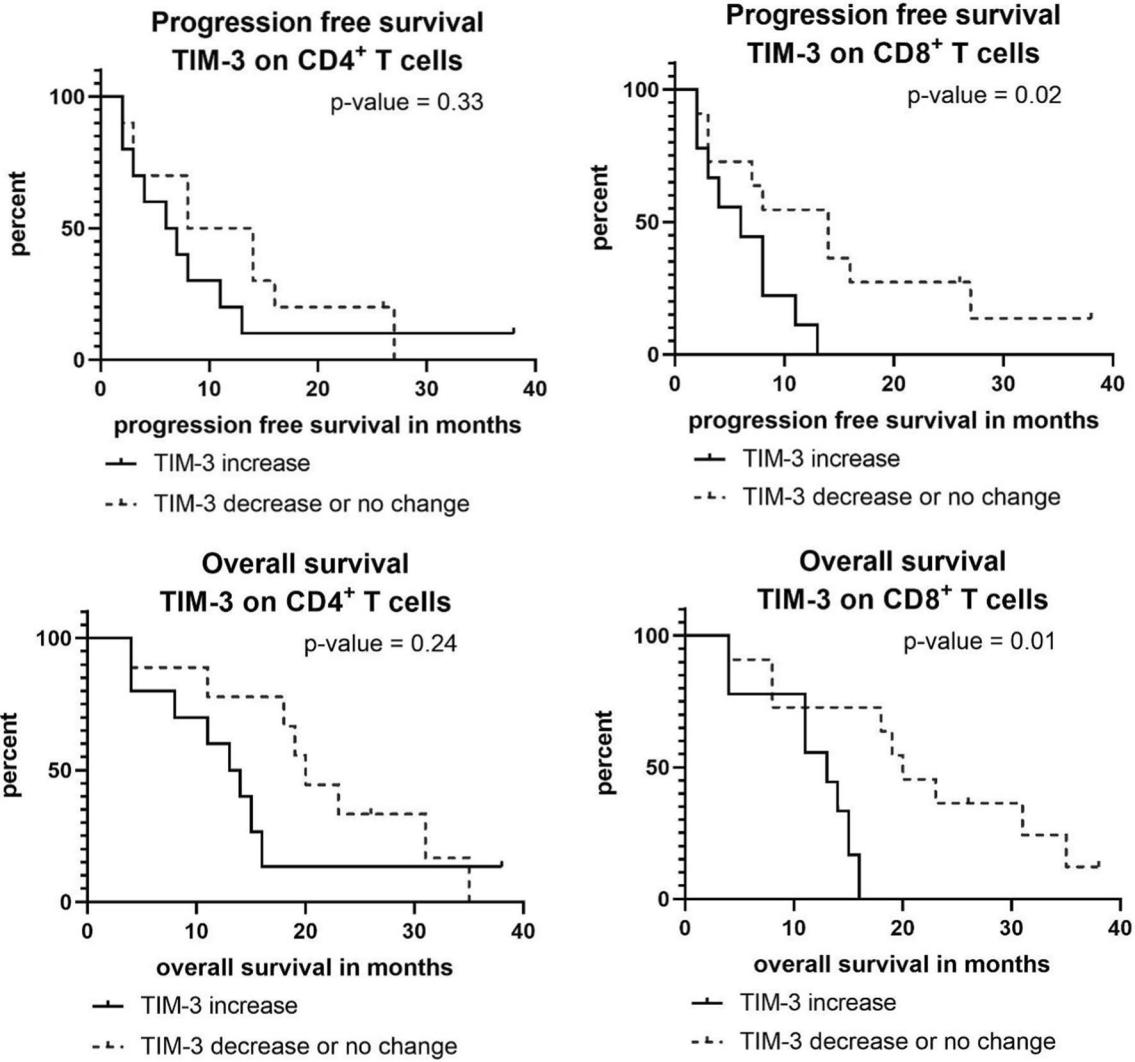
Progression-free and overall survival in patients with an increase or decrease/no change in TIM-3+/CD4+ or TIM-3+/CD8+ T cells over the first 30 days of chemotherapy

As displayed in Fig. 4, the increase of TIM-3+/CD4+ T cells was associated with a numerically shorter PFS (median 6.5 vs. 11.0 months, *p* = 0.331) and OS (median 13.5 vs. 20.0 months, *p* = 0.240). An increase of TIM-3+/CD8+ T cells showed a statistically significant outcome correlation: median PFS in the group with an increase of TIM-3+/CD8+ T cells was 6.0 months compared to 14.0 months in patients with a decrease or no change (*p* = 0.026); the corresponding median OS was 13.0 months and 20.0 months, respectively (*p* = 0.011).

## Discussion

Up to now, the effect of standard PDAC chemotherapy regimens on the patients’ immunological status (measured in circulating immune cells) remains widely unclear. However, these effects are of major scientific and clinical interest as from these observations novel options may arise regarding immunotherapeutic approaches. In addition, the impact of chemotherapy on the “fitness” of T cells and other lymphocyte subpopulations might have important implications for the design of chimeric antigen receptor (CAR) T cells trials in PDAC [15]. Different preclinical studies suggested that chemotherapeutic drugs might promote anti-tumor immune response by induction of immunogenic cell death and immune activation [16]. To date, however, no supporting evidence for this hypothesis from prospective translational or clinical studies exists. In this pilot study, we found a high variability in the dynamic expression of markers for Tregs and T-cell checkpoint molecules during chemotherapy (see Fig. 1). As no specific pattern was obvious, these findings do not support the idea of a general immune modulatory effect of FOLFIRINOX or gemcitabine/nab-paclitaxel in advanced PDAC. Recently, the PA.7 randomized phase II study promoted the notion that the synergistic effect of immune checkpoint blockade observed in preclinical models might not be relevant in PDAC patients: in that trial, the addition of a dual immune checkpoint inhibition (with durvalumab and tremelimumab) to standard gemcitabine/nab-paclitaxel did not improve outcome [17].

Previous reports on immunological effects of gemcitabine-based chemotherapy in PDAC have found changes in Tregs in peripheral blood as an indicator for an activation of the immune system [18]. Lui and co-workers demonstrated that a decrease of circulating Tregs in patients with PDAC under chemotherapy was associated with an improved OS [10]. There is valid evidence that an increase in Tregs is associated with immunosuppression that may enhance tumor progression and, therefore, Tregs may present a predictor for worse outcome [19]. Within our pilot study, we, however, did not find clear evidence for an impaired prognosis in patients with an increase of either CD4+ or CD8+ Tregs during chemotherapy (Fig. 2). Regarding the course of PD-1+ T cells during chemotherapy, only a non-significant trend between T-cell subsets and survival outcome was observed, with a poorer prognosis in patients with an increase of PD-1+ T cells under chemotherapy (Fig. 3). In line with those findings, Shen and colleagues recently reported a correlation between higher PDAC tumor stages and a high PD-1 expression on CD8+ T cells [20]. Also, the analysis of TIM-3 on CD4+ T cells showed a non-significant trend towards a worse prognosis in case of an increase of TIM-3+/CD4+ T cells. For an increase in TIM-3+/CD8+ T cells, a significant correlation with a shorter PFS and OS was found. This finding is supported by a recent study that reported an association of TIM-3+ T cells in PDAC tumor tissue with a worse prognosis [21].

A main limitation for our study arises from the rather low patient numbers that might also be a reason for the lacking statistical significance in some of the analysis on the prognostic impact of changes in circulating immune cells (e.g. PD-1+ T cells). However, in contrast to other groups that recently reported similar observations (no significant effect of systemic chemotherapy for solid tumors on circulating T cells subsets), we focused on only one disease entity with all patients having an advanced stage of disease and investigated changes during chemotherapy with two well-established regimens [22]. In that context, the current pilot study yielded interesting insights into alterations in circulating immune cells during the use of standard PDAC chemotherapy and a prospective validation in a larger multicenter cohort is recommended.
